# Early Alterations in Hippocampal Circuitry and Theta Rhythm Generation in a Mouse Model of Prenatal Infection: Implications for Schizophrenia

**DOI:** 10.1371/journal.pone.0029754

**Published:** 2012-01-06

**Authors:** Guillaume Ducharme, Germaine C. Lowe, Romain Goutagny, Sylvain Williams

**Affiliations:** Douglas Mental Health University Institute, McGill University, Department of Psychiatry, Montréal, Québec, Canada; University of Alberta, Canada

## Abstract

*Post-mortem* studies suggest that GABAergic neurotransmission is impaired in schizophrenia. However, it remains unclear if these changes occur early during development and how they impact overall network activity. To investigate this, we used a mouse model of prenatal infection with the viral mimic, polyriboinosinic–polyribocytidilic acid (poly I∶C), a model based on epidemiological evidence that an immune challenge during pregnancy increases the prevalence of schizophrenia in the offspring. We found that prenatal infection reduced the density of parvalbumin- but not somatostatin-positive interneurons in the CA1 area of the hippocampus and strongly reduced the strength of inhibition early during postnatal development. Furthermore, using an intact hippocampal preparation *in vitro*, we found reduced theta oscillation generated in the CA1 area. Taken together, these results suggest that redistribution in excitatory and inhibitory transmission locally in the CA1 is associated with a significant alteration in network function. Furthermore, given the role of theta rhythm in memory, our results demonstrate how a risk factor for schizophrenia can affect network function early in development that could contribute to cognitive deficits observed later in the disease.

## Introduction

Schizophrenia is a serious disease whose aetiology is known to include both genetic and environmental factors [1]. In a pioneer study Mednick and colleagues have observed that children in utero during the 1957 influenza epidemic had increased risk of developing schizophrenia [2]. The association between influenza exposure during pregnancy and schizophrenia symptoms has been confirmed by several studies (reviewed in [3]) and extended to many other pathogens including other viruses (HSV-2 [4], rubella [5], bacteria [6]), and even protozoan [7], [8] reviewed in[9]). These observations have led to the development of animal models of prenatal infection to better understand how infection during pregnancy contributes to the disease, and whether prenatal infection can be a useful model to understand schizophrenia. In these models, prenatal immune challenge was found to induce behavioural alterations related to schizophrenia (ex.: deficits in pre-pulse inhibition, latent inhibition, and amphetamine hypersensitivity [10] for review) and display developmental and pharmacological features concordant with the disease [11], [12]. Furthermore, at the biochemical level, several alterations in the GABAergic transmitter system have been observed after prenatal immune challenge: an increase in GABA_A_ α5 receptor [13], a decrease in GABA content [14] and a reduction of parvalbumin-immunoreactive cells in the prefrontal cortex [15]. These abnormalities are of particular interest as they are in agreement with those described on post-mortem schizophrenic brain [16]–[18].

Many neural circuits of the brain oscillate and synchronize over a wide range of frequencies. Such coordinated activity is believed to be crucial for higher cognitive functions like attention, memory and perception [19]. During oscillatory activity, a remarkable balance exists between excitatory and inhibitory synaptic activity [20], [21]. Given the evidence showing a severe GABAergic deficit in schizophrenia, it is not surprising that many types of oscillations have been found to be altered in this disease [22]. Accordingly, relatively new theories of schizophrenia postulate a direct link between GABAergic deficits, network oscillations and the behavioural abnormalities observed in schizophrenia especially cognitive impairments [23], [24].

Given the aetiological relevance of prenatal infection to schizophrenia and the evidence showing GABAergic deficits in this model, we examined whether prenatal immune challenge impairs the inhibitory network of the hippocampus and examined the synaptic changes involved early in development. We used systemic injection of poly I∶C early in pregnancy at gestational day 9, a well established model of prenatal infection, to induce a strong immune response in pregnant mice. We found that the resulting offspring showed a redistribution of inhibitory and excitatory synaptic strength as early as 15 days after birth which was associated with profound deficits in hippocampal theta oscillations in the CA1 region of the hippocampus. Overall, our results show how prenatal infection can affect the internal hippocampal circuitry and result in aberrant network activity.

## Methods

### Ethics statements

Experiments followed the guidelines established by the Canadian Council on Animal Care and were approved by the McGill University Animal Care Committee (protocol #5318).

### Animals

Timed-pregnant Swiss Webster mice (CFW; Charles River) were exposed to a 12 hour light/dark cycle with *ad libitum* access to food and water. On gestational day 9 the mice received a tail vein injection of either saline or poly I∶C (Polyinosinic-Polycytidylic acid sodium salt; 1 mg/kg). We used a concentration of 1 mg\kg since such treatment triggered a strong and transient immune reaction as observed by a severe hypothermia (change in body temperature measured at 2 hour post injection in control, −0.2±0.01°C, n = 34 vs. in poly I∶C, −1.50±0.15°C n = 32, t_(2,64)_ = 8.461, p<0.0001) that returned to baseline after 4 hours. In addition, this poly I∶C dose produced a large increase in IL-6 plasma level to 8.75 ng/ml at 3 hours in non-pregnant mice (Dr. Louise Harvey, personal communication) similar to previously reported data using 5 mg/Kg poly I∶C in a different strain of mice [25]. All experiments were performed on both male and female offspring and animals from a minimum of 4 different litters were used in each group for each experiments.

### Histology

At postnatal day 21 offspring from both control (8 mice) and poly I∶C (6 mice) treated mothers were intracardially perfused with 0.9% cold saline and then with 4% paraformaldehyde while under deep ketamine/xylazine anaesthesia. Brains were collected, post-fixed overnight in 4% PFA and cryoprotected in 30% sucrose before freezing. Coronal cryostat sections (25 µm thick) were cut and kept in a phosphate saline buffer (PBS) solution supplemented with 0.1% sodium azide until used. Immunohistochemistry was performed using a standard protocol with the following antibodies: α-parvalbumin mouse monoclonal IgG1 (1∶1000, Sigma), α-somatostatin rabbit polyclonal (1∶250, Santa Cruz Biotechnology), α-mouse IgG1 conjugated with Alexa Fluor 568 and α-rabbit conjugated with Alexa 488 (both from Molecular Probes). Sections were blocked in a gelatine solution (saline phosphate buffer, 2 g/L gelatine and 0.25% triton-X) and both primary and secondary incubations were done overnight at 4°C. All sections were processed together using the same stocks. One section every 100 µm was analysed and eight sections were evaluated per animal. The first section was chosen based on when the most rostral CA1 first appeared. This encompassed most of the dorsal portion of CA1. Photomicrographs were captured and all labelled cells were counted by an experimenter blind to the treatment. The cells were identified based on shape and size (roughly 10 to 20 µm in diameter) and cells with a large range of staining intensity were included. The counts were performed for each layer of CA1 individually and then divided by the area of that region. Image analysis was done using the ImageJ software. We cannot exclude that a change in cell density is due to a real reduction in the number of interneurons, a reduction in the expression level of the markers, or both.

### Electrophysiology

Mice (saline and poly I∶C 17 mice in each group, 16- to 21- day-old) were decapitated and brains were extracted and cooled in ice-cold sucrose ACSF solution containing (in mM): 252 sucrose, 24 NaHCO_3_, 10 glucose, 3 KCl, 2 MgSO_4_, 1.25 NaH_2_PO_4_, 1.2 CaCl_2_, 0.4 ascorbate. Coronal sections of the dorsal hippocampus were cut 300 µm thick. Slices remained in ACSF (the sucrose was replaced by 126 mM NaCl) and oxygenated with 95% O_2_/5% CO_2_ for 1 hour before recording. Recordings were performed in normal ACSF (2 mM CaCl_2_) at 30°C and constantly perfused at 3.0 ml/min. Patch pipettes for whole-cell recordings (Warner Instrument) had resistance between 2.8–4 MΩ. Intracellular solution contained (in mM) 144 K-gluconate (or 144 KCl for mini IPSC recording), 10 HEPES, 2 Na_2_ATP, 0.3 GTP, and 0.2 EGTA. Qx-314 (5 mM, Alomone) was added for evoked IPSC recordings. Junction potentials were not corrected for; they were −2 mV for recording miniIPSCs and −9 mV for evoked IPSC. All cells used for analysis had an access resistance below 30 MΩ.

Miniature IPSCs were recorded from hippocampal pyramidal cells in the presence of TTX (1 µM), AP-5 (10 µM) and DNQX (10 µM). A minimum of 300 events were recorded per cell from which 100 were randomly selected for analysis. To record antidromic IPSCs, CA1 pyramidal cells were patch-clamped and a 300 µA electrical stimulation was delivered (one every 15 s) through a monopolar tungsten electrode placed in the stratum oriens. The stimulating electrode was located approximately 250 µm distal to the recorded cells. In this recording configuration an IPSC that is dependent on both GABAergic and glutamatergic synaptic transmission is elicited. The the various components of this response were isolated as follow: the mixed IPSC was simply the baseline current before adding any blocker; the monosynaptic IPSC was measured as the current remaining after addition of DNQX (20 µM) and AP-5 (25 µM); the antidromic IPSC was calculated by subtracting the IPSC before and after addition of DNQX and AP-5; and lastly the NMDA dependent antidromic IPSC was obtained by the subtraction of the IPSC before and after AP-5 application. For these experiments, we used an ACSF containing 0.1 MgSO_4_ to increase the NMDA-mediated synaptic component of the IPSC. Slices were cut at the CA1/CA3 border to prevent antidromic activation of CA3. For all these experiments, only 1 cell was recorded per slice and no more than 2 from the same animal.

### Whole hippocampus preparation

Isolated intact hippocampi were prepared from 14- to 16-day-old mice (saline 7 mice and poly I∶C 6 mice) as previously described [26]. ACSF composition was the same as for slice except that KCl was raised to 4.0 mM. Field recordings were done with ACSF-filled patch pipettes (1–4 MΩ) placed at the CA1 stratum radiatum-pyramidale border in the middle of the longitudinal axis of the hippocampus and electrode placement was confirmed using dye injection. Only 1 hippocampus (randomly selected between the left and right) was used per animal. Signals were recorded with a differential AC amplifier (A-M systems), filtered online (0.1–500 Hz) and sampled at 5 kHz. Spectral analysis was done using the Chronux package [27] on MATLAB (MathWorks) and theta power was measured as the total power between 3 and 10 Hz. Statistical analysis was performed by t-test (unless otherwise noted) using the Prism 4 software (Graph Pad) and all data are shown as mean ± SEM. On the figures * denotes p<0.05, ** p<0.01 and *** p<0.001.

## Results

### Reduced inhibitory strength in CA1 after prenatal immune challenge

**Figure 1:**
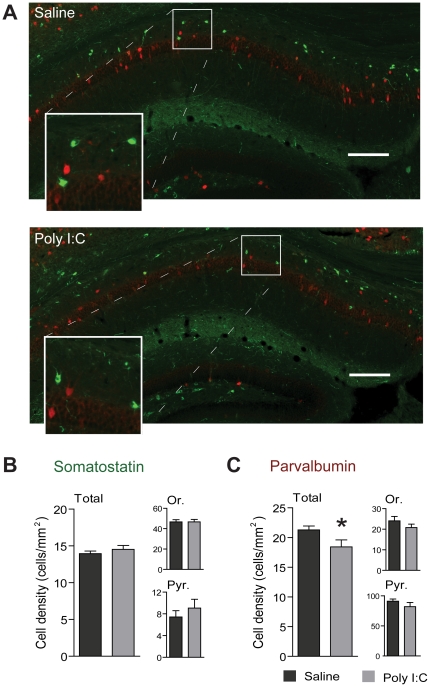
Reduced density of parvalbumin-expressing interneurons in CA1 caused by prenatal immune challenge. **A**) Photomicrograph of the CA1 subfield of the hippocampus from pups of dams treated with poly I∶C during pregnancy (bottom) and control (top). Scale bar 200 µm, in green: somatostatin, in red: parvalbumin. **B**) and **C**) Comparison of somatostatin- (B) and parvalbumin- (C) positive cell density between the poly I∶C and the saline group, for all the CA1 subfields (total), stratum oriens (Or.) or stratum pyramidale (Pyr.) (saline n = 8, poly I∶C n = 6).

To assess any early inhibitory deficit in the CA1 region of the hippocampus after prenatal immune challenge, we first estimated the number of interneurons using immunohistochemistry from postnatal day 21 mice. More specifically, we quantified the density of parvalbumin- and somatostatin-positive interneurons, two important populations of CA1 interneurons. Figure 1A shows representative photomicrographs of the resulting sections. Prenatal immune challenge did not affect the density of somatostatin-positive interneurons (Fig. 1B) but significantly decreased the density of parvalbumin cells in the CA1 area (saline 21.28±0.67 cell/mm^2^, n = 8 mice, poly I∶C 18.43±1.17 cell/mm^2^ , n = 6 mice, t_(2,12)_ = 2.26, p = 0.0436). However, it could not be determined whether this was due to changes in interneurons located in the stratum oriens (Or) or pyramidale (Pyr) as the densities in those subregions showed a trend but did not reach statistical significance (Fig. 1C).

**Figure 2:**
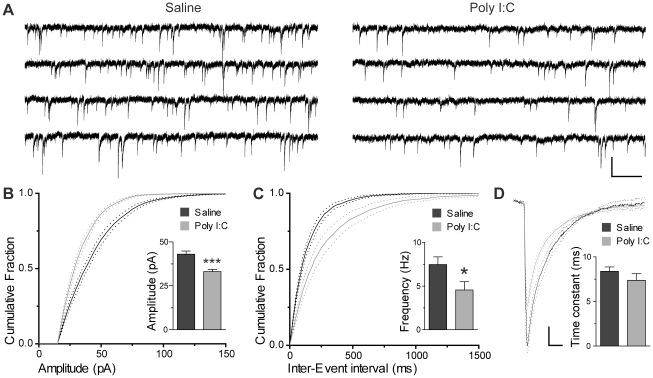
Prenatal immune challenge decreases the frequency and amplitude of miniIPSCs recorded in CA1 pyramidal cells. **A**) Representative miniIPSC recordings from animals prenatally exposed to saline (left) or poly I∶C (right). Scale bar 500 ms (horizontal) and 60 pA (vertical). **B**) Average cumulative amplitude histogram (± SEM) of miniIPSCs recorded from the saline and poly I∶C group. Inset: average mean IPSC amplitude (100 events averaged for each cell) recorded for each group. **C**) Average cumulative inter-event interval histogram (± SEM) of miniIPSCs recorded from saline and poly I∶C treated animals. Inset: average frequency of miniIPSCs of all cells recorded for each group. **D**) Average waveform of 100 mini events from each cell for both groups (± SEM), scale 5 pA (vertical) and 5 ms (horizontal). Inset: Average miniIPSCs time constant. n = 7 for both groups.

Given this reduction in parvalbumin-positive interneurons, we investigated whether this was translated into a decrease in the strength of inhibitory input to CA1 pyramidal cells. To accomplish this, we recorded miniature IPSCs in CA1 pyramidal cells using the whole cell patch-clamp technique in hippocampal slices *in vitro* recorded at postnatal day 16–21. Representative recordings of miniature IPSCs are shown in Fig. 2A. As expected, a significant decrease in the amplitude and frequency of miniature events were found in offspring after prenatal immune challenge (Fig. 2B and 2C). The amplitude reduction of mini IPSCs was apparent from the average cumulative amplitude histogram (Fig. 2B) as well as from the average amplitude (saline 43.00±1.82 pA n = 7 cells, poly I∶C 33.06±1.31 pA n = 7 cells, t_(2,12)_ = 4.43, p = 0.0008, see Fig. 2B inset). The frequency reduction of miniIPSCs was also apparent from the distribution of the inter-event intervals (Fig. 2C) as well as from the average frequency of events (saline 7.487±0.868 Hz, poly I∶C 4.558±0.956, t_(2,12)_ = 2.27, p = 0.0426, see Fig. 2C inset). No significant differences in the decay time constant were found between groups (Fig. 2D). Taken together, these results suggest that the decrease in the number of CA1 parvalbumin neurons is correlated with a significant functional decrease in GABAergic inhibition.

### Increased recruitment of local interneurons after prenatal immune challenge

**Figure 3:**
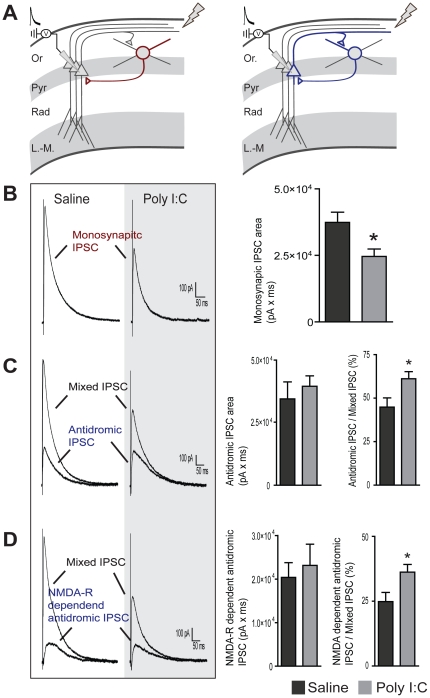
Prenatal immune challenge reduced monosynaptic IPSCs but increased recruitment of local inhibitory feedback interneurons. **A**) Recording configuration and diagram showing the two components of the mixed IPSC (composed of the monosynaptic IPSC from direct activation of local interneurons shown in red and the antidromic IPSC from feedback interneurons activated by CA1 recurrent projections shown in blue). **B**) monosynaptic IPSCs isolated by the addition of DNQX (20 µM) and AP-5 (25 µM). Left: representative recordings from both groups. Right: average monosynaptic IPSC area for both groups (n = 8 for each group). **C**) Isolation of the antidromic IPSC. Left: representative recording showing the mixed IPSC before the addition of DNQX (20 µM) and AP-5 (25 µM) and the isolated antidromic IPSC (IPSC sensitive to AP-5 and DNQX). Middle: average magnitude of the antidromic IPSC area from both groups. Right: fraction (in percent) of the mixed IPSC contributed by the antidromic IPSC (n = 8 for each group). **D**) Isolation of the NMDA receptor-dependent antidromic IPSC. Left: representative recording showing the mixed IPSC before the addition of AP-5 (25 µM) and the NMDA receptor-dependent antidromic IPSC. Middle: average magnitude of the NMDA receptor-dependent antidromic IPSC area from both groups. Right: fraction (in percent) of the mixed IPSC contributed by the NMDA receptor-dependent antidromic IPSC (n = 8 for saline and n = 7 for the poly I∶C treated group). Stimulation intensity was 300 µA and each trace displayed corresponds to the average of 5 consecutive responses.

We next aimed to further confirm the decrease in inhibitory strength that we observed after prenatal immune challenge using a different technique. To do so, IPSCs were elicited in CA1 pyramidal cells by the stimulation of the stratum oriens/alveus border. In this recording configuration, these IPSCs are comprised of a component arising from the direct activation of local interneurons (monosynaptic IPSC) and another component arising from the antidromic activation of CA1 pyramidal cells which then recruit local feedback interneurons through glutamate receptor activation (antidromic IPSC, see diagram in Fig. 3A). The combined monosynaptic and antidromic IPSCs were named mixed IPSCs. According to our previous results showing a strong decrease in inhibitory strength, we expect the monosynaptic and antidromic IPSCs to be reduced. As expected, the monosynaptic IPSCs (isolated by the addition of DNQX (20 µM) and AP-5 (25 µM)), was reduced in the poly I∶C group compared to controls (saline 3.74×10^4^±0.38 pA×ms, n = 8 cells, poly I∶C 2.46×10^4^±0.28 pA×ms, n = 8 cells, t_(2,14)_ = 2.69, p = 0.0175, Fig. 3B). However, when we examined the magnitude of the antidromic IPSC (current sensitive to DNQX and AP-5), no difference was found (saline 3.45×10^4^±0.68 pA×ms, n = 8 cells, poly I∶C 3.96×10^4^±0.42 pA×ms, n = 8 cells, t_(2,14)_ = 0.63, p = 0.54, Fig. 3C). These findings may suggest a compensatory increase in the local glutamatergic feedback on interneurons. To characterize this further, we expressed the result as the ratio of the antidromic IPSC over the mixed IPSC. In the absence of excitatory compensation, both components of this ratio will be equally decreased and, consequently, the ratio will be similar between both groups. However, we found that poly I∶C treatment increased this ratio (antidromic/mixed IPSC), suggesting a compensatory increase in the recurrent excitatory input on local interneurons (saline 44.9±5.3% vs. poly I∶C 61.1±4.1%, t_(2,14)_ = 2.42, p = 0.0297, Fig. 3C *left*).

We next investigated whether NMDA receptors were involved in this compensation. Despite the known reduction in inhibition, we found no difference in the antidromic IPSC dependent on NMDA receptors between both groups (saline 2.04×10^4^±0.34 pA×ms, n = 7 cells, poly I∶C 2.31×10^4^±0.49 pA×ms, n = 6 cells, t_(2,11)_ = 0.47, p = 0.65, Fig. 3D). In agreement with an increased NMDA receptor component, the NMDA-dependent antidromic/mixed IPSC ratio was increased by prenatal immune challenge (saline 24.9±3.5% n = 8 cells, vs. poly I∶C 36.2±2.9% n = 7 cells, t_(2,13)_ = 2.42, p = 0.0310, Fig. 3D *left*). These results suggest that compensatory changes may occur in the glutamatergic-dependent recruitment of local interneurons and suggest that part of this compensation is mediated by NMDA receptors.

### Prenatal immune challenge decreases CA1 network activity in the theta frequency range

Our results show many alterations in the internal circuitry of the CA1 of the hippocampus after prenatal immune challenge. We next investigated whether these GABAergic and glutamatergic synaptic changes were associated with altered network activity. Specifically, we used an intact hippocampal preparation *in vitro* [26] to investigate whether theta rhythm generation in the CA1 area was affected. This whole hippocampal preparation has several advantages; first, it is able to self-generate theta oscillations without any pharmacological stimulation which avoids potential confounds arising from drugs normally used to generate theta *in vitro* and second, the theta rhythm obtained is entirely generated within CA1 [26].

**Figure 4:**
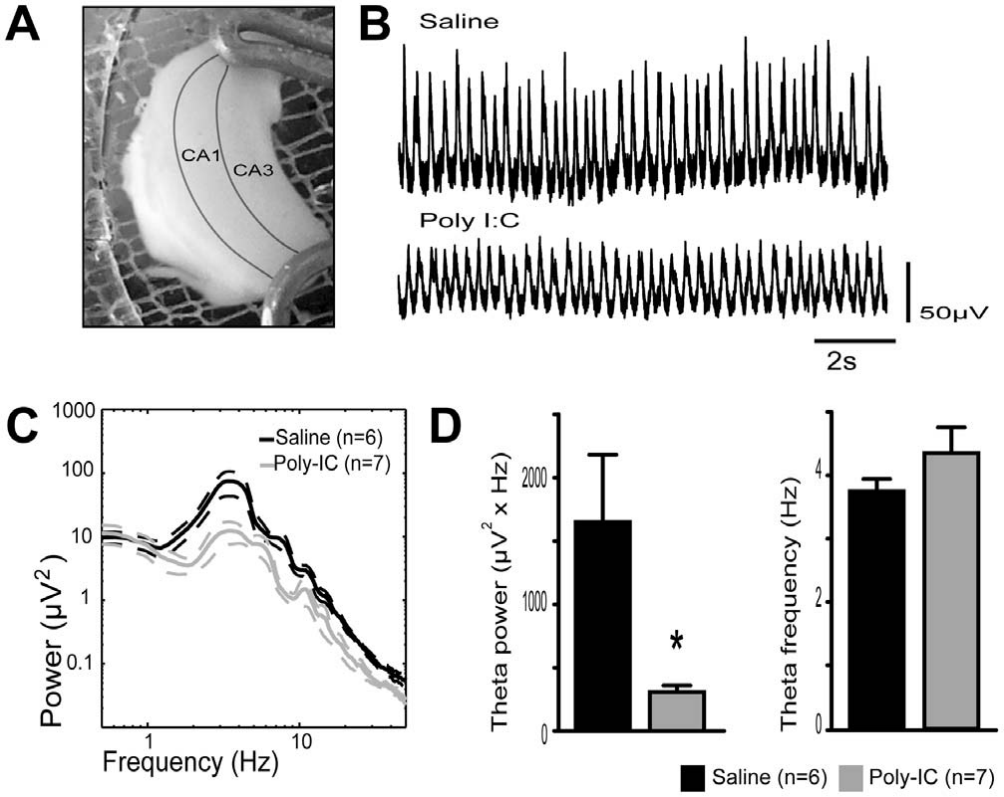
Reduction of CA1 theta power in the hippocampus after prenatal immune challenge. **A**) Photo of the intact hippocampal preparation with CA1 and CA3 outlined. **B**) Representative recording of spontaneous oscillatory activity in the whole hippocampal preparation *in vitro*. **C**) Average power spectrum (± SEM). Notice in both cases the peak power at around 4 Hz, corresponding to theta rhythm. **D**) The average power and peak frequency of the spontaneous self-generated theta oscillation recorded in the whole hippocampal preparation from poly I∶C- and saline-treated animals. For this experiment n = 6 in the saline and n = 7 in the poly I∶C-treated group.

In the whole hippocampal preparation done on postnatal day 14–16 mice, we recorded spontaneous theta activity in the CA1 area and compared the power and peak frequency between controls (n = 7) and poly I∶C-treated animals (n = 6). Figure 4A shows representative theta field recordings obtained from the two groups. We used multi-taper analysis to extract spectral data from the local field potential recordings (Fig. 4B). The peak theta frequency of the oscillation was not different between the control and the poly I∶C treated group (control 3.75±0.1936 Hz, poly I∶C 4.357±0.4285 Hz, t_(2,11)_ = 1.28, p = 0.150). However, a large decrease in theta band power was found after prenatal immune challenge (saline 1650±531 µV^2^ vs. poly I∶C 306±54 µV^2^, t_(2,11)_ = 2.73, p = 0.0195, Fig. 4C).

## Discussion

There is compelling evidence that the GABAergic inhibitory system is affected in schizophrenia and may contribute to the cognitive deficits associated with the disease. However, the period at which these changes occur and how they impact the overall inhibitory and excitatory network functions remains unclear. In the present study we tested the hypothesis that prenatal immune challenge precociously impairs the GABAergic network causing local excitatory-inhibitory imbalance affecting theta rhythm generation.

Alteration of the GABAergic network has been repeatedly observed in the neocortex of schizophrenics [24]. Interestingly, some of these changes appear specific to some subtypes of interneurons, as parvalbumin cells in particular have been shown to be affected in the prefrontal cortex [28] and in the hippocampus [16]. A decrease in parvalbumin-positive interneurons has also been shown in several animal models of schizophrenia [29]–[31] including in prefrontal cortex and hippocampus after a late pregnancy (gestational day 17) prenatal immune challenge [15]. In the later study, a decrease in parvalbumin cells was not observed in their early pregnancy (gestational day 9) poly I∶C model in the dorsal hippocampus, which appears inconsistent with the present study. However, Meyer and colleagues [15] assessed parvalbumin cells number in all the hippocampal subfields together (CA1, CA3 and dentate gyrus) which may have masked the small decrease we observe in CA1. Alternatively, several studies suggest that a reduction in parvalbumin expression rather than a loss of parvalbumin cells *per se* may be more characteristic of schizophrenia. This was demonstrated in an animal model using ketamine administration [32] as well as in post-mortem schizophrenic brains [33]. Accordingly, we cannot rule out the possibility that the decrease in parvalbumin cells we observed here may be due a reduced expression of parvalbumin and a failure to detect these cells with weak parvalbumin expression in the Poly I∶C treated animals. In their study, Meyer and colleagues [15] used a method that can result in large amplification of weak immunoreactivity and as such might not have detected the effect of a reduced parvalbumin expression, which may further explains the discrepancy with the results presented here. Nevertheless, the decrease in parvalbumin-positive interneurons observed here after an early challenge (gestational day 9) is in good agreement with both animal models of the disease and human data. However, our results show that the reduction in parvalbumin-immunoreactive interneurons in the CA1 area occurs at or before 3 weeks of age, suggesting that inhibitory loss is a precocious event in this model.

Importantly, we have demonstrated that this loss in interneurons was accompanied by a strong decrease in functional inhibition within the CA1 region of the hippocampus. Indeed, we found that the frequency and amplitude of miniature IPSCs were reduced in pyramidal cells. The decrease in frequency is consistent with a decrease in the number of inhibitory synapses on pyramidal cells which may be due to the loss of parvalbumin interneurons. As for the decrease in amplitude of the miniIPSCs, it is likely due to a decrease in postsynaptic GABA_A_ receptors or a decrease in GABA vesicular content. It is noteworthy that a decrease in GABA content has been found using a neurochemical assay in the hippocampus of animals from the same model we used [14] and that mRNA for GAD67 (glutamic acid decarboxylase, one of the two isoform of the GABA-synthesizing enzyme) has been found to be reduced in the hippocampus of schizophrenic patients [17].

Given the decrease in functional inhibition we observed in CA1, we expected the antidromic evoked IPSC to be also decreased. Interestingly, antidromic IPSCs were found to be similar in size thus providing evidence of a compensatory increase in excitatory input on interneurons. This increase appears to be mediated partly by an NMDA receptor-dependent component, while a contribution of AMPA receptors could not be ruled out. A measurement of excitatory AMPA/NMDA input in the remaining interneurons will provide definite evidence of this phenomenon. We suggest that after prenatal immune challenge the CA1 network may be more dependent on glutamatergic synaptic transmission to maintain GABAergic inhibition. In accordance with our results, there is evidence that animals prenatally treated with poly I∶C early in pregnancy are more sensitive to NMDA antagonists, as they show increased MK-801 (an NMDA-receptor antagonist) induced locomotion [34]. In a network that is more dependent on glutamatergic synaptic transmission, factors that impair glutamatergic-dependent synaptic transmission should have a larger impact on the network, providing a possible mechanism for synergistic interaction between risk factors. Synergistic interactions between prenatal infection and genetic predispositions in schizophrenia has been shown both in human [35] as well as in animal models [36]. Furthermore, such interactions, specifically at the level of NMDA receptors, may be relevant for the disorder as several genetic risk factors for schizophrenia, like Dysbindin, reelin and DISC-1, have been shown to alter NMDA-receptor function in rodents [37]–[39]. This convergence of GABAergic and glutamatergic deficits at the level of interneuron recruitment has been proposed by others as a key mechanism that integrate the deficits in these two neurotransmitter systems in schizophrenia [40].

Recently we have shown that spontaneous theta rhythm can be recorded in the isolated intact hippocampus in the CA1, without pharmacological stimulation, which shares many characteristics with *in vivo* theta [26]. *In vivo*, it is well established that hippocampal theta oscillations arise from the simultaneous inputs from several cortical and subcortical structures such as the entorhinal cortex and medial septum, respectively [41]. Our recent results suggest that the hippocampus contains intrinsic theta oscillators in the CA1 [26] and that these external oscillators may modulate intrinsic theta generators. The presence of a robust theta generator within CA1 *in vitro* is thus well suited to determine how network changes in excitatory and inhibitory balance alters CA1 theta because it excludes any other changes coming from outside CA1. In that study, we had shown that CA1 theta was most likely the result of pyramidal cells spiking on the rebound of IPSPs and, consequently, that any change in IPSP size and/or kinetics would directly affect pyramidal cell spiking and lead to changes in theta. Thus, it is likely that a reduction in IPSC size in pyramidal cells after prenatal poly I∶C treatment would cause a reduction in rebound firing and a reduction in theta power. These results are also consistent with data showing that theta oscillations are dependent on proper inhibitory tone [41] and that parvalbumin-positive interneurons play an important role [42], [43].

Theta oscillations are essential to learning and memory (reviewed in [44]. For example, theta power during encoding predicts retrieval success in human [45], [46]. In rodents, abolition of hippocampal theta causes spatial memory deficits [47]. Therefore a reduction in theta rhythm after prenatal immune challenge may contribute to the learning and memory impairment found in this model of prenatal infection [34]. In addition, a reduced capacity of the hippocampus to engage in theta oscillation would likely contribute to the decrease synchronization that has been observed between the hippocampus and prefrontal cortex in this model [48].

In conclusion, the present study suggests possible mechanisms through which prenatal infection may contribute to the pathophysiology of schizophrenia: 1) alterations in parvalbumin GABAergic interneurons and functional inhibition occur early after prenatal infection and may pave the way to later anomalies in the disease; 2) this early insult may increase the dependence of the network on glutamatergic synaptic transmission potentially increasing the action of other risk factors; and, 3) inhibitory-excitatory imbalance are associated with changes in theta oscillations which may contribute to the altered theta synchronisation and deficit in memory processes observed in this model. Because these changes occur early in development, they may constitute some of the early events in a chain of biochemical and physiological alterations leading to the behavioural abnormality associated with the disease.
